# The *Drosophila* G protein-coupled receptor, GulpR, is essential for lipid mobilization in response to nutrient-limitation

**DOI:** 10.1371/journal.pgen.1011982

**Published:** 2025-12-12

**Authors:** Daniela Barraza, Xiang Ding, Lauren Findley, Zihuan Wang, Bat-Erdene Jugder, Paula I. Watnick

**Affiliations:** 1 Division of Infectious Diseases, Boston Children’s Hospital, Boston, Massachusetts, United States of America; 2 Biological and Biomedical Sciences Program, Harvard Medical School, Boston, Massachusetts, United States of America; 3 Department of Pediatrics, Harvard Medical School, Boston, Massachusetts, United States of America; Walter and Eliza Hall Institute of Medical Research, AUSTRALIA

## Abstract

Enteroendocrine cells (EECs) of the intestinal epithelium are major regulators of metabolism and energy homeostasis. This is mainly due to their expression and secretion of enteroendocrine peptides (EEPs). These peptides serve as hormones that control many aspects of metabolic homeostasis including feeding behavior, intestinal contractions, and utilization of energy stores. Regulation of EEP production and release depends largely on EEC-exclusive G protein-coupled receptors (GPCRs) that sense nutrient levels. Here we report the characterization of a GPCR expressed principally in EECs, which we have named GulpR due to its role in the response to nutrient stress. We show that GulpR regulates transcription of the EEP Tachykinin (Tk) and that both GulpR and Tk are essential for the transcriptional response that promotes survival of nutrient limitation. Oral infection with *V. cholerae* also activates expression of GulpR, Tk, and lipid mobilization genes. However, Tk does not play a role in regulation of lipid mobilization genes during infection and does not impact survival. Our findings identify a role for GulpR and Tk in survival during starvation and suggest that, although starvation and infection result in significant mobilization of energy stores, the signal transduction systems that regulate the metabolic response to each are distinct.

## Author summary

Humans and other animals, including *Drosophila*, catabolize dietary nutrients such as polysaccharides, lipids, and proteins into simple sugars, fatty acids, and amino acids, respectively, to generate the energy that fuels essential cellular processes like cell division, ion transport, muscle contraction, and more. The ability to adapt to changes in nutrient availability and energy demand is therefore crucial for homeostasis and survival. Nutrient scarcity and the energy demands of an immune response against pathogenic infection require utilization of the body’s own lipid and glycogen stores. This adaptive response largely relies on the ability of the intestine to sense and respond to a variety of stimuli, including microbes and dietary nutrients. Here, we have characterized a *Drosophila melanogaster* receptor that is expressed in a rare intestinal cell type with endocrine function. We report that this receptor regulates production of peptide hormones that are known to impact metabolic homeostasis and discover that one of these peptide hormones is crucial for utilization of systemic lipid stores under nutrient limitation but not infection stress. Our findings therefore indicate that lipid mobilization in response to nutrient limitation and infection are regulated via different mechanisms.

## Introduction

Maintenance of metabolic homeostasis is crucial for the health span and life span of living organisms. This requires matching food consumption and catabolism to the energy expended. This balance is achieved in part through the action of gut-derived peptide hormones produced in enteroendocrine cells (EECs) [1]. EECs comprise the largest endocrine organ in the body, secreting more than 20 different enteroendocrine peptides (EEPs) [2–4]. EEPs can act in a paracrine fashion to regulate nutrient absorption, gut motility, epithelial renewal and other processes within the intestinal epithelium or in an endocrine fashion to regulate appetite, satiety, metabolism, and energy expenditure. Appropriate EEP expression and secretion relies on the ability of EECs to sense nutritional status and nutrient intake via systemic and intestinal signals, respectively. This is achieved, in large part, by the action of cell-autonomous G protein-coupled receptors (GPCRs). GPCRs are cell membrane-associated receptors that activate a signal transduction cascade in response to binding small molecules or peptides. In EECs, GPCRs respond to nutrients, metabolites secreted by the intestinal microbiota, bile acids, neuropeptides, or other molecules by regulating EEP expression and exocytosis-mediated secretion [3,5,6].

*Drosophila melanogaster* has been used extensively as a model organism for the study of gastrointestinal (GI) processes as its intestine shares functional homology with the mammalian GI tract [7,8]. The adult *Drosophila* intestinal epithelium is subdivided into foregut, midgut, and hindgut sections [9]. The *Drosophila* midgut epithelium is composed of intestinal stem cells and two mature cell types: enterocytes (ECs), and enteroendocrine cells (EECs). While ECs are most abundant, EECs account for 5–10% of cells in the intestinal epithelium [9]. As in humans, *Drosophila* EEPs play crucial roles in regulating both intestinal physiology and systemic metabolism [10–12].

The *Drosophila* midgut can be subdivided into three regions (anterior, middle, posterior), exhibiting unique functional properties [9,13]. The microbiota of the fly resides mainly in the anterior midgut (AMG), which is also the compartment responsible for metabolism of complex macromolecules such as polysaccharides, fats, and proteins. Here we focus on regulation of the EEP Tachykinin (Tk), which is essential for innate immune signaling and lipid homeostasis in the AMG [10,14,15]. Tk has also been shown to impact lipid reserves in the *Drosophila* fat body, which is the main site of energy storage in the fly and functionally analogous to human adipose tissue and the liver [10,16–18].

To survive starvation or infection, animals must mobilize stores to meet their energy requirements [16–18]. Here we describe an orphan GPCR we have named GulpR that is specifically expressed in EECs, regulates Tk expression in response to nutrient stress and infection, and is essential for survival of starvation but not infection. These findings suggest that, while the responses to starvation and infection require considerable energy expenditure, distinct signaling pathways participate in generating the metabolic response to each. Thus, the type of energy demand encountered determines the signaling pathway utilized to mobilize lipids.

## Results

### A GulpR mutant displays decreased numbers of Tk-expressing (Tk+) cells in the AMG and PMG but increased lipid storage in the AMG only

In a previous RNA-seq experiment using *Vibrio cholerae*-infected whole flies, we noted a positive correlation between the intestinal levels of the short-chain fatty acid (SCFA) acetate during infection and expression of CG32547, a GPCR previously shown to be EEC-specific [19–21]. We renamed this GPCR GulpR for its action in the setting of nutrient limitation, which we elucidate here. GulpR has two predicted transcripts RC and RD, which encode identical polypeptides. These transcripts differ only in the length of their 3’ untranslated regions. Both consist of 7 exons of which 6 contain coding sequences (S1 Fig) [22].

**Fig 1:**
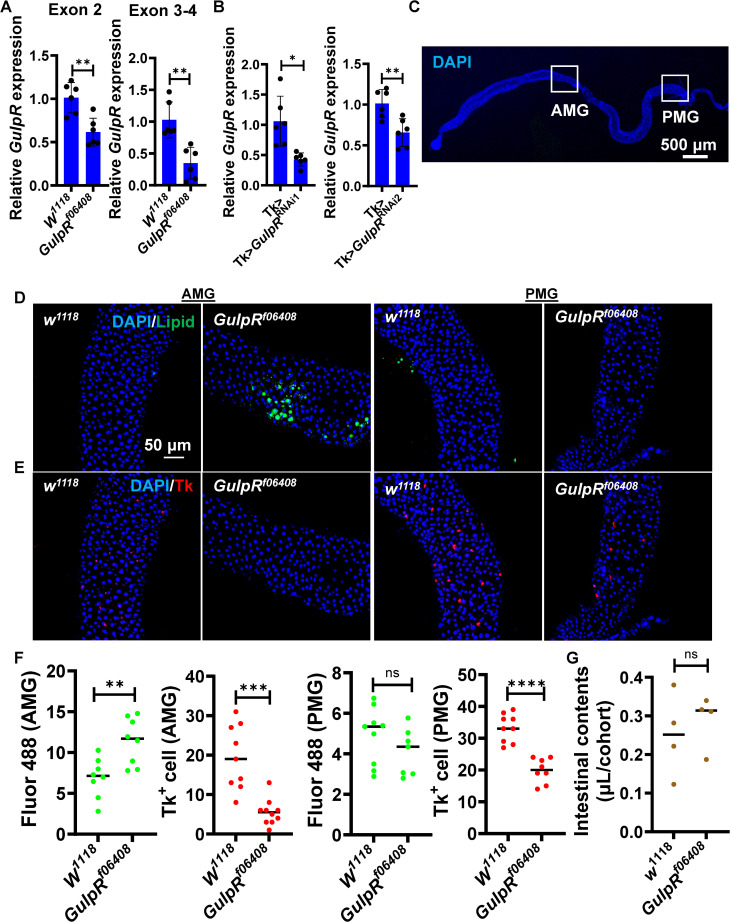
A *GulpR* mutant displays increased AMG lipid storage and decreased Tk+ EECs. **(A)** qRT-PCR analysis of *GulpR* transcription in the intestines of control *w*^*1118*^ or *GulpR*^r06408^ flies using primers upstream (Exon 2) and bridging (Exon 3-4) the transposon insertion. **(B)** qRT-PCR analysis of *GulpR* transcription in the intestines of control Tk> or Tk>*GulpR*^RNAi^ flies. Two RNAi’s were used (RNAi1: PTRIP.JF03036 and RNAi2: PTRIP.HMS05678). The mean of six biological replicates is shown. Error bars represent the standard deviation. A student’s t test was used to assess significance with the exception of the first RNAi comparison where a Welch’s t test was used. **(C)** Micrograph of the *Drosophila* gut. Boxed areas show the regions of the gut from which the AMG and PMG images were taken. Scale bar 500 µM. **(D)** Representative fluorescence images showing DAPI and BODIPY(Lipid) staining in the anterior midgut (AMG) and posterior midgut (PMG) of the indicated fly genotypes. **(E)** Representative immunofluorescence images showing DAPI and Tk staining in the anterior midgut (AMG) and posterior midgut (PMG) of the indicated fly genotypes. Scale bar, 50 μM. **(F)** Quantification of total fluorescence and Tk+ EECs in the AMG and PMG of the indicated fly genotypes. The mean of at least six intestines is shown. A student’s t test (Bodipy staining) or Welch’s t test (Tk immunofluorescence) was used to assess significance. **(G)** Contents of the intestines of fly cohorts of the indicated genotypes. The mean of four groups of five flies is shown. A student’s t test was used to assess significance. **** p < 0.0001, *** p < 0.001, ** p < 0.01, * p < 0.05, ns not significant.

Because acetate prevents lipid accumulation in the *Drosophila* AMG by increasing Tk expression, we questioned whether GulpR might be involved in regulating Tk expression and/or release [14]. To test this hypothesis, we first characterized an available *GulpR* transposon-insertion mutant fly (*GulpR*^f06408^) [23]. This transposon is located at the beginning of intron 3 and, therefore, interrupts both the RC and RD transcripts (S1 Fig). Adult female flies between 4–7 days of age were used to confirm that *GulpR* transcription was reduced in the intestines of *GulpR*^f06408^ flies as compared with control *w*^*1118*^ using primers both upstream of and bridging the transposon insertion (Figs 1A and S1). We then questioned whether *GulpR* was expressed in Tk+ EECs. To test this, we crossed a Tk> driver shown to principally target Tk+ EECs with two independent *GulpR*^RNAi^ lines, *GulpR*^JF03036^ (RNAi1) and *GulpR*^HMS05678^ (RNAi2) [10,24]. Transcription of *GulpR* in the intestines of Tk>*GulpR*^RNAi1^ and Tk>*GulpR*^RNAi2^ flies was significantly decreased compared with Tk> flies (Fig 1B). This confirms that *GulpR* is present in Tk+ cells. We then quantified Tk+ EECs and lipid accumulation in *w*^*1118*^ and *GulpR*^*f06408*^ flies in the regions of the AMG and PMG shown in Fig 1C. We observed an accumulation of lipids in the AMG of the *GulpR* mutant but not the PMG. This was accompanied by a reduction in the number of Tk+ EECs in both the AMG and PMG (Fig 1D-1F). To confirm that this was not the result of increased food intake, we performed a feeding assay that showed no difference in intestinal food contents (Fig 1G). We conclude that GulpR regulates Tk expression and/or secretion in both the AMG and PMG, but lipid accumulation only in the AMG.

### *GulpR*^RNAi^ in Tk+ EECs decreases intestinal Tk expression and increases lipid accumulation

**Fig 2:**
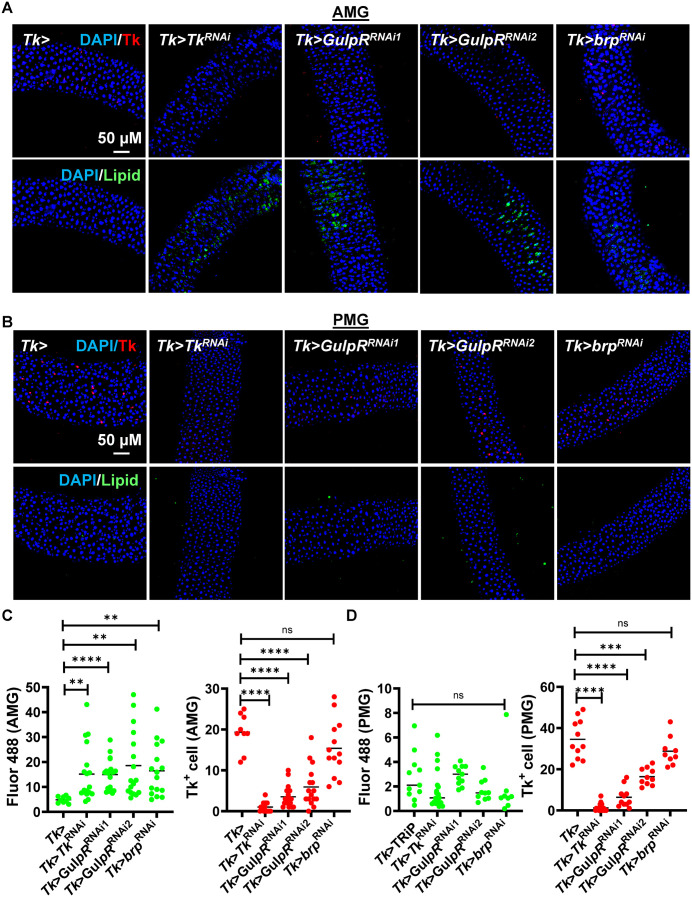
*GulpR*^RNAi^ in Tk+ EECs decreases Tk expression in the AMG and PMG but increases lipid accumulation in the AMG only. **(A and B)** Representative fluorescence images showing DAPI and **(A)** BODIPY(Lipid) staining or **(B)** Tk immunofluorescence in the anterior midgut (AMG) and posterior midgut (PMG) of the indicated fly genotypes. Scale bar, 50 μM. Quantification of total fluorescence and Tk+ EECs in the **(C)** AMG and **(D)** PMG of the indicated flies. The mean of at least six intestines is shown. For lipid quantification, a one-way ordinary ANOVA was used to assess significance. For quantitation of Tk+ EECs, a Welch’s ANOVA with a Dunnett’s T3 multiple comparisons test was used. **** p < 0.0001, ** p < 0.01, * p < 0.05, ns not significant.

To determine whether cell-autonomous GulpR expression is required to maintain Tk levels and lipid homeostasis in the *Drosophila* intestine, we compared the intestinal phenotype of Tk> flies with Tk>*GulpR*^RNAi1^ and Tk>*GulpR*^RNAi2^ flies. Like the *GulpR* mutant, Tk>*GulpR*^*RNAi1*^ and Tk>*GulpR*^*RNAi2*^ flies exhibited an increase in lipid storage in the AMG and a decrease in the number of Tk+ EECs in the AMG and PMG (Fig 2). Because this had not previously been reported, we questioned whether accumulation of lipids in the AMG represented region-specific Tk regulation of lipid homeostasis or a direct action of GulpR [10]. To explore this, we compared lipid accumulation and Tk staining in the intestines of Tk > *Tk*^RNAi^ flies with that in Tk>*GulpR*^RNAi1^, Tk>*GulpR*^RNAi2^, and Tk> intestines. In fact, Tk+ EECs were decreased in the intestines of Tk > *Tk*^RNAi^ flies beyond that observed for Tk>*GulpR*^RNAi1^ and Tk>*GulpR*^RNAi2^ flies but lipid accumulation was observed only in the AMG (Fig 2). These results indicate that GulpR regulates Tk expression in the AMG and PMG, but Tk regulates lipid utilization only in the AMG. We have not yet uncovered a role for Tk in the PMG. We also used an antibody targeting the general EEC marker prospero (pros) to quantify total EEC’s in the AMG and PMG of Tk> and Tk>*GulpR*^RNAi1^ flies to evaluate the possibility that decreased Tk+ EECs was the result of an overall decrease in EECs. As shown in S2 Fig, total numbers of pros+ EECs were not significantly different in the AMG and PMG of Tk> and Tk>*GulpR*^RNAi1^ flies. A small decrease was noted for Tk > *Tk*^RNAi^ flies. Because EEC Tk expression in Tk > *Tk*^RNAi^ flies is decreased beyond that of Tk>*GulpR*^RNAi1^ flies, this suggests that large decreases in Tk expression may impact EEC development. To establish that regulation of Tk expression by GulpR does not represent a developmental phenotype, we used a temperature-sensitive pros driver to conditionally drive activation expression of *GulpR*^RNAi1^ and *Tk*^RNAi^ in adulthood. This yielded a similar phenotype similar to that observed for Tk> *GulpR*^RNAi1^ and *Tk*^RNAi^ (S3 Fig).

GPCRs may regulate EEP secretion as well as expression. Therefore, we examined the possibility that a block in secretion might result in negative feedback on expression leading to a decrease in Tk+ cells. Bruchpilot (brp) is an exocytosis factor that is essential for neuropeptide release from vesicles at neural synapses [25–27]. While brp has not been studied in the intestine, it is highly transcribed in EECs but not other intestinal cell types [20]. Given the similar processes of vesicle release from EECs and neural synapses, we hypothesized that *brp*^RNAi^ might perform a function in EECs similar to that at neural synapses, and, thus, block Tk release. Tk > *brp*^RNAi^ resulted in lipid accumulation in the AMG but had no impact on the number of Tk+ EECs (Fig 2). These findings support our hypothesis that brp is essential for Tk release from EECs and, furthermore, suggest that a block in Tk release does not lead to a decrease in *Tk* expression. Our results establish a role for *GulpR* in activating Tk expression but do not rule out an additional role for GulpR in Tk secretion.

### GulpR activates expression of additional EEPs produced by Tk+ EECs

**Fig 3:**
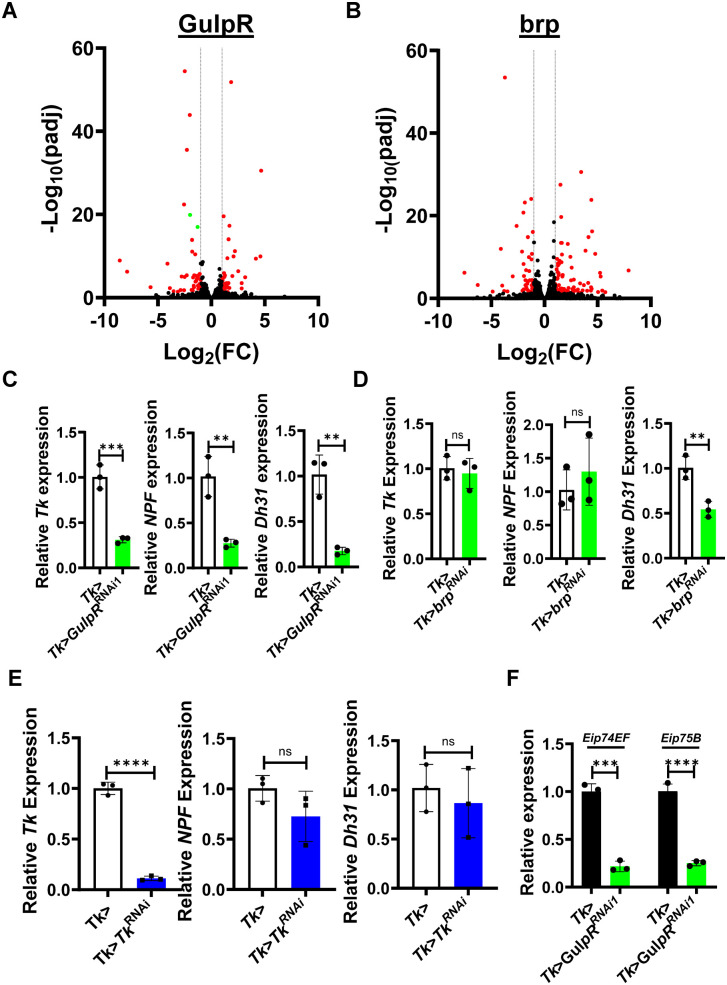
*GulpR*^RNAi^ in Tk+ EECs alters expression of innate immune genes and EEPs. **(A and B)** Volcano plots of gene expression ratios derived from RNAseq analyses of **(A)** Tk>*GulpR*^RNAi1^/Tk> or **(B)** Tk > *brp*^RNAi^/Tk. Dotted lines indicate thresholds of 2-fold differences in transcription, while red dots represent genes that were significantly differentially regulated. Green dots represent EEPs. **(C-E)** qRT-PCR analysis of genes encoding enteroendocrine peptides expressed by Tk+ cells in the intestines of the indicated fly lines. **(F)** qRT-PCR analysis of the ecdysone-regulated genes *Eip74EF* and *Eip75B* in the indicated fly lines. The mean of biological triplicates is shown. Error bars indicate the standard deviation. A student’s t test was used to assess significance. **** p < 0.0001, *** p < 0.001, ** p < 0.01, ns not significant.

To further differentiate regulation of EEP transcription and secretion, we conducted an RNA sequencing experiment comparing the intestinal transcriptomes of Tk>*GulpR*^*RNA1i*^ and Tk > *brp*^*RNAi*^ flies to that of Tk> flies. Using a threshold of 2-fold change and a padj of 0.05, 82 genes were differentially regulated by *GulpR*^RNAi1^ with 41 increasing in transcription and 41 decreasing (Fig 3A and S1 Table). Using the same criteria, *brp*^RNAi^ differentially regulated 124 genes with 78 increased and 46 decreased (Fig 3B and S2 Table). Twenty-nine genes were similarly regulated by the two, suggesting overlapping but distinct functions. Genes whose transcription was uniquely decreased in the intestines of Tk>*GulpR*^*RNAi1*^ flies included *Tk* as well as two additional EEPs expressed in the same EEC subtype: *Neuropeptide F* (*NPF*) and *Diuretic hormone 31* (*Dh31*). In contrast, none of these reached our threshold in Tk > *brp*^*RNAi*^ flies. These results were confirmed by qRT-PCR (Fig 3C and 3D). We measured a 1.8-fold decrease in *Dh31* transcription in the intestines of Tk > *brp*^RNAi^ flies. A similar pattern was observed in the RNAseq experiment, suggesting that the action of brp or a product released from Tk+ cells regulates *Dh31* transcription. To establish that Tk does not regulate transcription of *Dh31* and *NPF* directly, we measured transcription of these genes in Tk> and Tk > *Tk*^RNAi^ flies. While *Tk*^RNAi^ did decrease transcription of *Tk* significantly, no change in transcription was observed for *Dh31* and *NPF* (Fig 3E). We conclude that GulpR directly regulates EEP expression.

We have previously uncovered signaling pathways that regulate EEP expression via ecdysone activation of IMD signaling in Tk+ EECs [14,15,28]. To investigate the role of ecdysone signaling and the IMD pathway in GulpR regulation we measured transcription of the ecdysone signaling genes *Eip74EF* and *Eip75B* as well as the IMD pathway genes *PGRP-LC*, *Rel*, and *Dpt*, in Tk> and Tk>*GulpR*^RNAi1^ flies. As shown in Figs 3F and S4, *GulpR*^RNAi^ decreased transcription of both ecdysone-regulated and IMD pathway genes. Therefore, GulpR likely regulates EEPS via ecdysone-mediated IMD activation through an as yet unelucidated pathway.

### Intestinal GulpR and Tk are essential for regulation of lipid utilization during starvation

**Fig 4:**
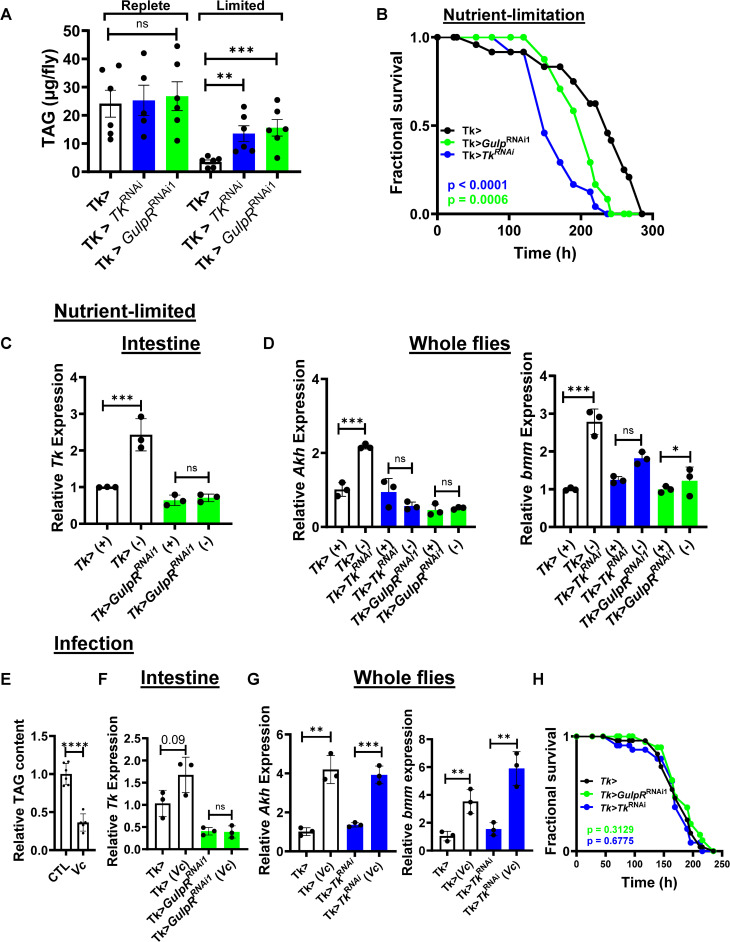
GulpR regulates lipid mobilization during starvation but not infection. **(A)** Quantification of systemic triglycerides (TAG) in flies of the indicated genotypes after 48 h of maintenance in nutrient-replete (+) and nutrient-limited (-) conditions. The mean of six biological replicates is shown. Error bars represent the standard deviation. A log-normal ordinary one-way ANOVA with Dunnett’s multiple comparisons test was used to assess significance. **(B)** Survival of nutrient-limitation over time for flies with the indicated genotypes. Thirty flies were tested per condition. Log-rank analysis was used to assess significance. **(C)** qRT-PCR analysis of transcription of *Tk* in the intestines of flies with the indicated genotypes under fed (+) and nutrient-limited (-) conditions for 72 h**.** **(D)** qRT-PCR analysis of *Akh* and *bmm* in whole flies with the indicated genotypes under fed (+) and nutrient-limited (-) conditions for 72 h**. (E)** Quantification of systemic triglycerides (TAG) in *yw* flies after 72 hours of infection with *V. cholerae* (*Vc*). The mean of at least five biological replicates is shown. Error bars represent the standard deviation. A student’s t test was used to assess significance. **(F)** qRT-PCR analysis of *Tk* transcription in the intestines of flies with the indicated genotypes under uninfected or *V. cholerae*-infected (*Vc*) conditions. **(G)** qRT-PCR analysis of *Akh* and *bmm* transcription in whole flies with the indicated genotypes under uninfected or infected (*Vc*) conditions. The mean of biological triplicates is shown. Error bars represent the standard deviation. A student’s t test was used to assess significance. **(H)** Survival of flies with the indicated genotypes over time after oral infection with *V. cholerae*. Thirty flies were tested per condition. Log-rank analysis was used to assess significance. **** p < 0.0001, *** p < 0.001, ** p < 0.01, * p < 0.05, ns not significant.

During long term starvation and infection, organisms use lipid stores to satisfy their energy requirements. We hypothesized that GulpR might play a role in one or both of these processes. We found that both starvation and infection increased GulpR expression (S5 Fig). We then characterized Tk > , Tk>*GulpR*^RNAi1^, and *Tk*^RNAi^ flies under nutrient-replete and nutrient-limited conditions. Total lipid stores were similar in Tk > , Tk > *Tk*^RNAi^, and Tk>*GulpR*^RNAi1^ flies under nutrient-replete conditions (Fig 4A). As expected, lipid stores decreased in response to nutrient limitation. However, under nutrient-limited conditions, lipid stores were significantly greater in *Tk*^RNAi^ and Tk>*GulpR*^RNAi1^ flies as compared with control flies. Furthermore, numbers of Tk+ cells increased in the setting of starvation for Tk> flies in the AMG only (S6 Fig). This was accompanied by a small but significant decrease in intestinal lipid accumulation. In contrast, Tk>*GulpR*^RNAi1^ and *Tk*^RNAi^ flies showed no increase in Tk+ cells during starvation. Additionally, accumulated lipids in the intestines of Tk>*GulpR*^RNAi1^ and *Tk*^RNAi^ flies were not expended as they were for Tk> flies. This suggests that both GulpR and Tk are essential for appropriate mobilization of lipid stores during starvation. We hypothesized that appropriate use of lipid stores during starvation might be essential for survival of nutrient stress. In fact, we found that Tk>*GulpR*^RNAi1^ and Tk > *Tk*^RNAi^ flies succumbed to starvation more rapidly than Tk> flies (Fig 4B).

Because GulpR regulates *Tk* transcription and both play a role in survival of starvation, we questioned whether GulpR might regulate *Tk* transcription in response to nutrient-limitation. To test this, we measured *Tk* transcription in the intestines of Tk> and Tk>*GulpR*^RNAi1^ flies. As shown in Fig 4C, nutrient limitation significantly increased intestinal *Tk* transcription in Tk> flies, but this increase was not observed in Tk>*GulpR*^RNAi1^ flies. These findings were consistent with the quantification of Tk+ EECs shown in S6 Fig. The glucagon homolog Adipokinetic hormone (Akh) is responsible for mobilization of energy stores from the fat body in response to starvation, and brummer (bmm) is the main TAG lipase in the fly [29,30]. We, therefore, hypothesized that GulpR and Tk might activate transcription of these two genes in response to nutrient limitation. To test this, we measured systemic transcription of *Akh* and *bmm* in Tk > , Tk > *Tk*^RNAi^, and Tk>*GulpR*^RNAi1^ under both nutrient-replete and nutrient-limited conditions. Nutrient limitation resulted in transcriptional activation of *Akh* and *bmm* in Tk> flies but not in Tk > Tk^*RNAi*^ and Tk>*GulpR*^RNAi1^ flies (Fig 4D). This suggests that GulpR directly regulates *Tk* under conditions of starvation resulting in activation of *Akh* and *bmm* transcription, which are predicted to appropriately mobilize lipid stores.

### Intestinal GulpR and Tk are dispensable for lipid utilization during infection with the intestinal pathogen *Vibrio cholerae*

Pathogenic infection activates the host immune response in a process that is energetically costly [31,32]. Similar to starvation, flies orally infected with *V. cholerae* lost significant amounts of their TAG stores (Fig 4E). Having established that GulpR regulates intestinal *Tk* and that Tk regulates systemic *Akh* and *bmm* during starvation, we reasoned that it might play a similar role during oral infection with the intestinal pathogen *Vibrio cholerae* [33]. *V. cholerae* infection modestly increased transcription of *Tk* in the gut and EECs in the AMG, and the increases were blocked by Tk>*GulpR*^RNAi1^ (Figs 4F and S7). However, Tk did not control systemic *Akh* and *bmm* transcription in the setting of infection (Fig 4G). Furthermore, neither GulpR nor Tk played a role in survival of infection (Fig 4H). We conclude that, while GulpR regulates Tk expression in the setting of infection, increases in systemic *Akh* and *bmm* transcription are regulated via a distinct pathway. As a result, GulpR plays no role in survival of infection.

## Discussion

EECs primarily function in expression and secretion of EEPs, which modulate local and systemic physiology. Regulation of EEP production relies on the ability of EECs to sense intestinal nutrient status, which is mainly achieved via nutrient transporters and GPCRs. In addition to nutrients, EEC GPCRs can sense and respond to other factors present in the intestine, including inflammatory cytokines, gut peptides, and neurotransmitters. Previously, we identified the EEC transporter Tarag, which functions as an acetate importer to activate expression of Tk [15]. Here, we characterize GulpR, an EEC GPCR that regulates expression of EEPs, including Tk, in the setting of metabolic stress.

Although we have not identified the ligand that activates GulpR signaling here, two studies suggest the type of ligands that might interact with GulpR. GulpR has been reported to be closely related to the Neuropeptide Y and prolactin-releasing peptide receptors [34]. Neuropeptide Y and prolactin-releasing peptide are both peptide hormones produced mainly in the vertebrate brain with some expression in the intestine [35–37]. In addition, a phylogenetic study suggested that GulpR is closely related to a *Drosophila* orphan GPCR CG12290, which is a predicted octopamine receptor [38]. We, therefore, hypothesize that the endogenous ligand of GulpR is either a neuropeptide or a neurotransmitter.

EEPs are important regulators of metabolic homeostasis. There are two Tk receptors that are expressed in the gut, TkR99D and TkR86C. Tk interacts with its cognate receptor TkR99D on enterocytes to prevent accumulation of lipids in the intestine [10]. In the current study, we found that during starvation expression of the *Drosophila* EEP Tk increases and is essential for transcriptional activation of two critical lipid utilization genes *Akh* and *bmm*. One possibility is that Tk acts directly on the endocrine cells of the corpora cardiaca (CC), which are the main producers of Akh [39]. These cells express the Tk receptors TkR86C and TkR99D, and it has been reported that addition of Tk to the corpora cardiaca (CC) of *Locusta migratoria* ex vivo stimulates Akh release [40,41].

Much like starvation, infection by the enteric pathogen *V. cholerae* induces GulpR-dependent upregulation of *Tk.* However, the significant increases in *Akh* and *bmm* transcription that are activated by infection are present even in the absence of Tk and neither Tk nor GulpR promotes survival of infection. Our work, therefore, suggests that there is redundancy in the signaling pathways that activate *Akh* and *bmm* transcription to regulate lipid mobilization. While lipid mobilization is regulated mainly by the GulpR/Tk pathway in the setting of starvation, during infection, GulpR and Tk play minor roles and other signaling pathways dominate.

## Materials and methods

### *Drosophila* husbandry and strains

Fly lines used in this study were fed standard Bloomington recipe fly food containing 16.5 g/L yeast, 9.5 g/L soy flour, 71 g/L cornmeal, 5.5 g/L agar, 5.5 g/L malt, 7.5% corn syrup, 0.28% Tegosept (w/v), and 0.4% propionic acid. Flies were raised in a 12 hr day-night cycle incubator at 25°C. Adult female flies between 4–7 days of age were used in all experiments. The control lines *TRiP* (BL36303) and the *GulpR* mutant stock (*GulpR*^*f06408*^:BL18976) were obtained from the Bloomington *Drosophila* Stock Center (BDSC). The following RNAi fly stocks were also obtained from the BDSC: *GulpR*^RNAi1^ (BL28621), *GulpR*^RNAi2^ (BL67868)*, Tk*^RNAi^ (BL2500), and *brp*^RNAi^ (BL25891). The *w*^1118^ line was a lab stock. The Tk*-*Gal4 and the pros^ts^-Gal4 driver lines were kind gifts from Norbert Perrimon and Bruce Edgar, respectively. For the latter temperature-sensitive driver, flies were maintained at 21 °C until adulthood and transferred to 29 °C for 5–8 days to activate transcription.

### Measurement of food intake

5-7 days female flies were collected from vials containing standard fly food. These flies were starved for 2 hours and then transferred to standard medium containing 2% (w/v) FD&C Blue #1 dye (Sigma-Aldrich, 861146). Flies were harvested after 24 hours, and groups of 5 flies were homogenized in 100μL PBS. After centrifugation at 16,000g for 5min to remove particulate matter, absorbance of the supernatant was measured at 629 nm on a SpectraMax iD5 plate reader. Flies fed food alone were processed similarly. The A_629_ of these control supernatants were subtracted from those of the test supernatants. Serial dilutions of the dye were used to generate a standard curve.

### Starvation-resistance

Three cohorts of 10 flies were placed and maintained in vials containing an autoclaved cellulose plug infused with 3mL of 1x PBS. For survival, the number of viable flies was recorded twice daily until the number of viable flies was 0 for all genotypes. For RT-qPCR assays, the flies were kept in the vials containing 1x PBS for 72 hours and then processed for the appropriate experiment.

### *V. cholerae* infection

The quorum sensing-competent *V. cholerae* 01 El Tor strain C6706 (C6706 str 2) was used for fly infections. *V. cholerae* was grown in LB broth supplemented with 100 µg/mL streptomycin at 27°C. Three cohorts of 10 flies were orally infected with a 10-fold dilution of an overnight culture of *V. cholerae* in LB broth as follows. Flies were placed in vials containing an autoclaved plug infused with 3mL of the bacterial suspension and allowed to ingest it continuously. The number of viable flies was recorded twice daily.

### *Drosophila* RNA extraction, RNA-sequencing, and RT-qPCR

RNA was isolated from the intestines or whole bodies of 4–7-day old female flies that were kept in standard fly food, starved for 72 hours by placing in vials containing cellulose plugs infused with 3 mL of 1x PBS, or infected with quorum sensing-competent *V. cholerae* 01 El Tor strain C6706 (C6706 str2) for 72 hours. Total RNA was isolated from 10-15 fly intestines or 5–8 whole flies per replicate using TRIzol reagent (Thermo Fisher Scientific 15596026) and the Direct-zol RNA Miniprep plus kit (Zymo Research R2070). A minimum of three biological replicates per condition/genotype were performed. For RNA sequencing analysis, the RNA was submitted to the Molecular Biology Core Facilities (MBCF) at the Dana Farber Cancer Institute (DFCI) for next-generation sequencing (NGS) library preparation, sequencing, and analysis (https://mbcf.dana-farber.org/totalrnaseq.html). Libraries were prepared using Roche Kapa mRNA HyperPrep strand specific sample preparation kits from 200ng of purified total RNA according to the manufacturer’s protocol on a Beckman Coulter Biomek i7. The finished dsDNA libraries were quantified by Qubit fluorometer and Agilent TapeStation 4200. Uniquely dual indexed libraries were pooled in an equimolar ratio and shallowly sequenced on an Illumina MiSeq to further evaluate library quality and pool balance. The final pool was sequenced on an Illumina NovaSeq X Plus targeting 40 million 150 bp read pairs per library. Sequenced reads were aligned to the UCSC hg38 reference genome assembly and gene counts were quantified using STAR (v2.7.3a) [42] and Salmon [43]. Differential gene expression testing was performed by DESeq2 (v1.22.1) [44]. RNAseq analysis was performed using the VIPER snakemake pipeline [45].

For qPCR, cDNA was synthesized from 500ng of total RNA using the iScript cDNA synthesis kit (Bio-Rad 1708891). qPCR of target gene transcripts was performed using the iTaq Universal SYBR Green Supermix (Bio-rad 1725121) on either a StepOnePlus Real-Time PCR system (Applied Biosystems) or a QuantStudio 5 Real-Time PCR system (Thermo Fisher Scientific). Quantification cycle values (Cq) were obtained and used to calculate target gene transcription normalized to Actin. Primers used in this study are listed in S3 Table.

### Immunofluorescence

Fly intestines were dissected in 1x PBS, fixed in 4% paraformaldehyde (PFA) solution (4% PFA in 1x PBS- 0.1%Tween 20 (PBT)) for 20 minutes, and washed three times for 10 minutes with 1x PBT. For Tk-immunofluorescence experiments, intestines were left in blocking solution (PBT + 0.1% Triton X-100 (Sigma-Aldrich 9002-93-1) + 2% BSA (Sigma-Aldrich 9048-46-8)) for 1 hour, and then in Rabbit anti-Tk primary antibody solution (blocking solution + 1:500 anti-Tk antibody) overnight at 4°C. The next day the guts were washed three times with PBT for 10 minutes, followed by incubation in staining solution #1 (blocking solution + 1:1000 DAPI (Invitrogen D1306) + 1:500 Alexa 594-conjugated Goat anti-rabbit secondary antibody (Thermo Fisher Scientific A11012)) for 2 hours. For pros staining, a monoclonal anti-pros antibody (DHSB MR1A) was used in a 1:20 concentration with a secondary Alexa 488-conjugated Goat anti-mouse antibody (Thermo Fisher Scientific A11001) in a 1:500 dilution. For lipid staining, the intestines were incubated in staining solution #2 (PBT + 1:1000 DAPI (Invitrogen D1306) + 1:1000 BODIPY 493/503 (Invitrogen D3922)) for 2 hours after the initial fixing and washing steps. After three 10-minute washes in PBT, the guts were mounted in Vectashield antifade mounting medium (Vector Laboratories H-1000–10) and imaged using a Zeiss LSM 980 confocal microscope and a 40x oil objective. Unless otherwise noted, all steps were done at room temperature. Tk+ cells were quantified manually. Lipid accumulation was assessed using ImageJ (FIJI) to quantify total BODIPY fluorescence. The corrected total cell fluorescence (CTCF) was calculated by dividing total BODIPY fluorescence by the total area and subtracting background fluorescence from this measurement. A minimum of six fly intestines per genotype were evaluated for quantification of Tk-expressing cells and BODIPY fluorescence.

### Triglyceride quantification

Where indicated, flies were starved for 48 hours or infected with *V. cholerae* for 72 hours prior to the measurement. Three cohorts of 5 flies were washed with cold 1x PBS in a 9-well glass plate. The flies were then homogenized in 100µL of cold 1x PBS-0.05% Tween using a plastic pellet pestle (Fisher Scientific) and a Fisherbrand Pellet Pestle Cordless Motor (Fisher Scientific) or a TissueLyser III (Qiagen) set to 25 Hz for 6 mins. For the TissueLyser method, wells included one 3 mm autoclaved glass bead. The homogenate was kept on ice until a heat-inactivation step involving incubation at 70 °C for 10 min. After heat-inactivation, 20µL of 1x PBS-0.05% Tween buffer or 20µL of triglyceride reagent (Sigma-Aldrich T2449) were added to 20µL of the homogenate and the mixture was incubated at 37 °C for 1 hour. The samples were centrifuged at maximum speed for 3 min and 20µL of the supernatant were transferred to a clear-bottom 96-well plate. These samples were treated with 100µL of free glycerol reagent (Sigma-Aldrich F6428). Absorbance was measured at 540nm using a SpectraMax ABS absorbance microplate reader (Molecular Devices). Relative triglyceride (TAG) levels were calculated by subtracting the absorbance of samples treated with buffer (free glycerol) from samples treated with triglyceride reagent (free glycerol + fatty acids), dividing by the number of flies in each cohort (5), and normalizing to the control genotype.

### Gene maps, quantification and statistical analysis

GulpR architecture shown in S1 Fig was obtained from FlyBase (release FB2025_04) via the JBrowse genome browser [22]. All data was graphed and analyzed using GraphPad Prism 10.0 software. Numbers of biological replicates are shown and reported in the Figure Legends. Measurements shown in each graph represent the mean values of biological replicates, and the error bars represent ± the standard deviation. A student t-test, Welch’s t test, ordinary one-way ANOVA, Welch’s ANOVA, or a Log-rank test was used to determine significance as noted in the Figure legends.

## Supporting information

S1 Fig Architecture of GulpR/CG32457. (PDF)

S2 Fig Knockdown of *GulpR* in Tk+ EECs does not change total EEC numbers. (PDF)

S3 Fig Knockdown of *GulpR* after eclosion decreases Tk+ EECs in the AMG and PMG and increases lipid accumulation in the AMG. (PDF)

S4 Fig Knockdown of *GulpR* in Tk+ EECs decreases intestinal ecdysone and IMD signaling. (PDF)

S5 Fig Both infection and starvation increase expression of GulpR. (PDF)

S6 Fig Knockdown of *GulpR* and *Tk* in Tk+ EECs blocks an increase in Tk+ EEC and utilization of lipids in the AMG during starvation. (PDF)

S7 Fig Knockdown of *GulpR* in Tk+ EECs blocks an increase in Tk+ EECs during *V. cholerae* infection. (PDF)

S1 Table Genes differentially regulated in RNAseq of uninfected Tk>GulpR RNAI flies as compared with Tk> driver only flies (Tk > RNAi/Tk>). Driver-only flies were crossed to a Trip control fly line (y sc v).
(XLSX)

S2 Table Genes differentially regulated in RNAseq of uninfected Tk > *brp*^RNAI^ flies as compared with Tk> driver only flies (Tk > RNAi/Tk>). Driver-only flies were crossed to a Trip control fly line (y sc v).
(XLSX)

S3 Table Primers used in qRT-PCR experiments. (XLSX)
