# Supplementary material for: The Drosophila G protein-coupled receptor, GulpR, is essential for lipid mobilization in response to nutrient-limitation
Source: PLoS Genet. 2025 Dec 12;21(12):e1011982. doi: 10.1371/journal.pgen.1011982 (PMC12711087; doi:10.1371/journal.pgen.1011982)
Supplement: S1 Fig — (PDF) [file pgen.1011982.s001.pdf]

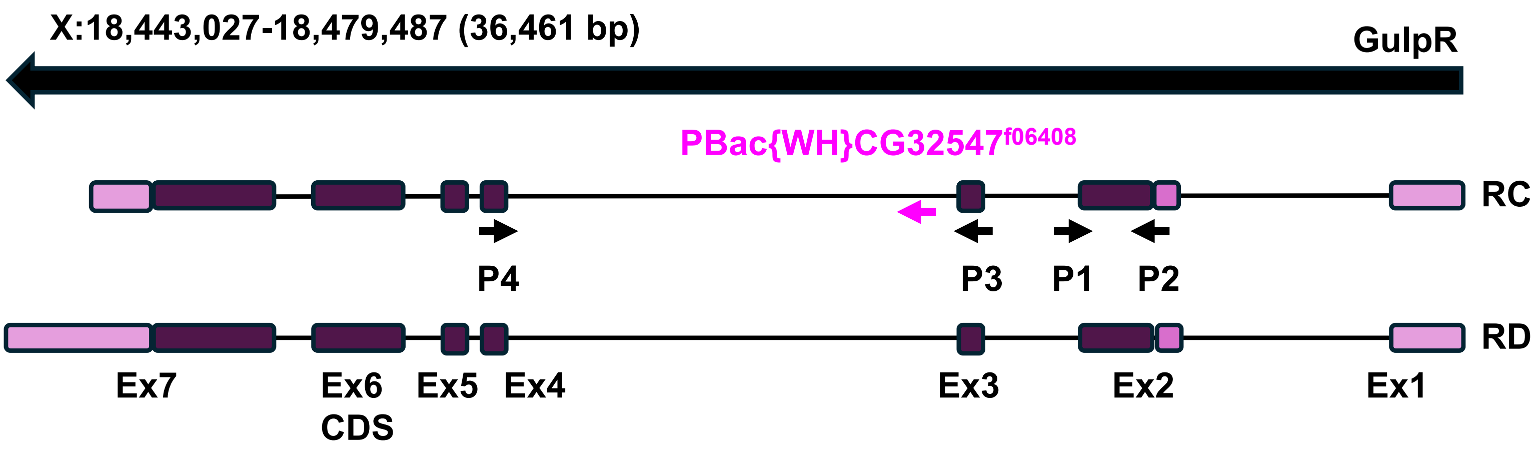

S1 Fig: Architecture of GulpR/CG32457. Schematic of the architecture of GulpR. Dark purple boxes represent coding sequences (CDS) and light purple boxes represent untranslated regions (UTR). GulpR has two transcripts RC and RD that differ only in the length of their 3ʹ UTRs. Both consist of 7 exons (Ex). The position of the transposon insertion mutant PBac{WH}CG32547^{f06408} is indicated by a pink arrow. The primers used to amplify GulpR upstream of and bridging the transposon insertion are indicated by P1-P2 and P3-P4, respectively.
