# Supplementary material for: The Drosophila G protein-coupled receptor, GulpR, is essential for lipid mobilization in response to nutrient-limitation
Source: PLoS Genet. 2025 Dec 12;21(12):e1011982. doi: 10.1371/journal.pgen.1011982 (PMC12711087; doi:10.1371/journal.pgen.1011982)
Supplement: S2 Fig — (PDF) [file pgen.1011982.s002.pdf]

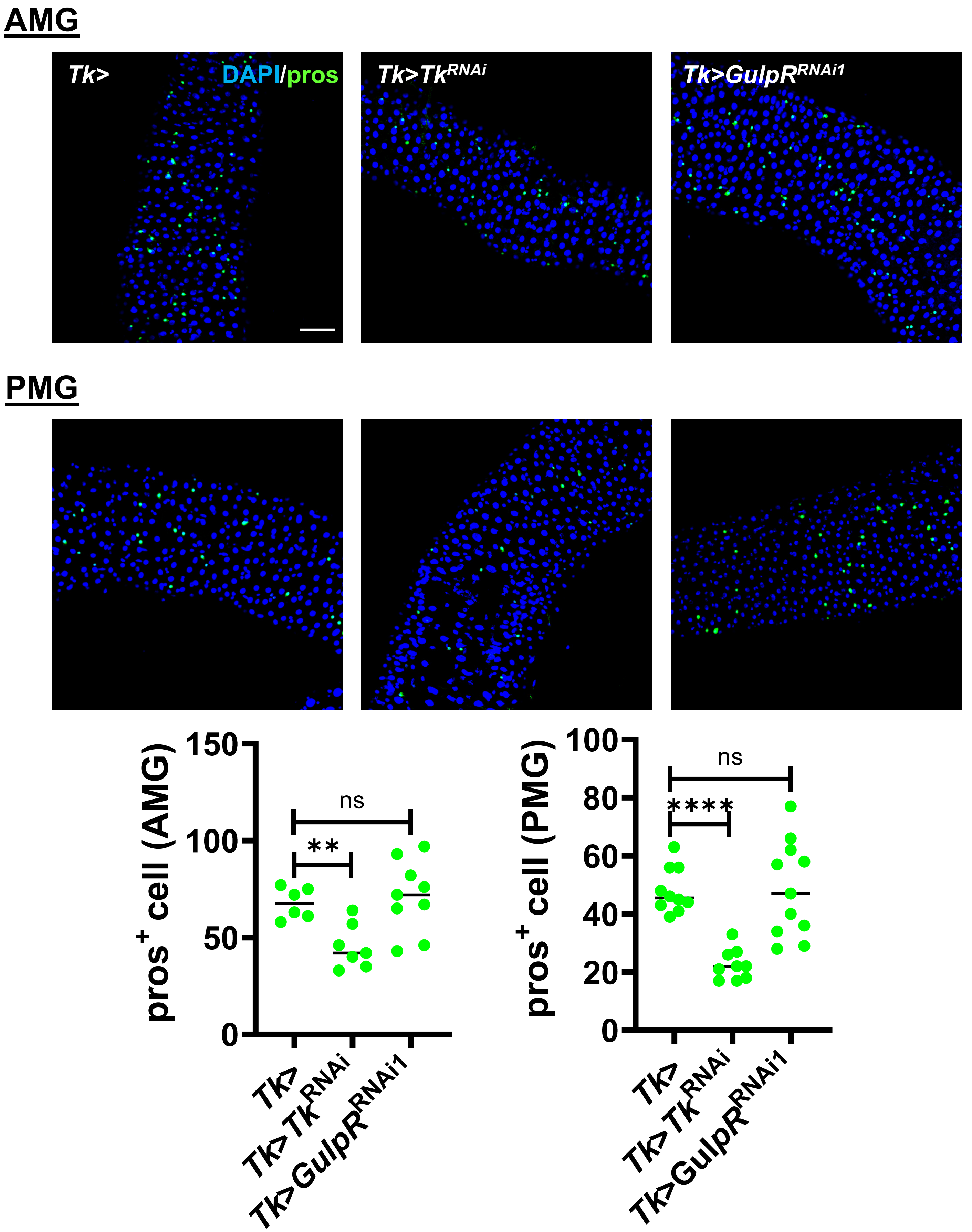

S2 Fig: Knockdown of *GulpR* in Tk+ EECs does not change total EEC numbers. Representative micrographs and quantification of pros+ cells in the AMG and PMG of flies of the indicated genotype. The mean of a minimum of 9 intestines is shown. Scale bar 50 µM. Significance was calculated using a Welch's ANOVA with Dunnett's T3 multiple comparisons test.. **** p<0.0001, ** p<0.01, ns not significant.
