# Supplementary material for: The Drosophila G protein-coupled receptor, GulpR, is essential for lipid mobilization in response to nutrient-limitation
Source: PLoS Genet. 2025 Dec 12;21(12):e1011982. doi: 10.1371/journal.pgen.1011982 (PMC12711087; doi:10.1371/journal.pgen.1011982)
Supplement: S3 Fig — (PDF) [file pgen.1011982.s003.pdf]

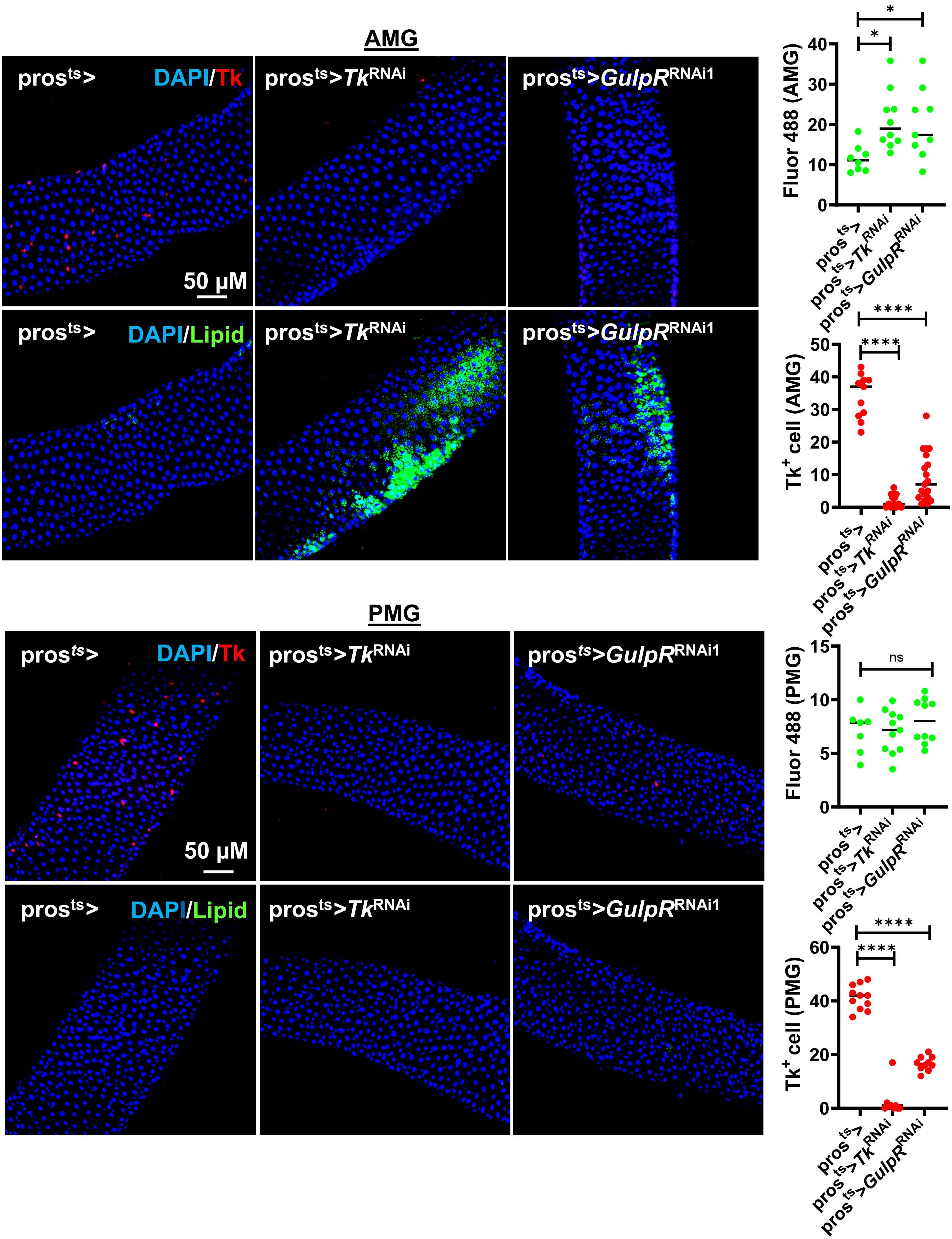

AMG
pros^ts> DAPI/Tk
pros^ts>Tk^RNAi
pros^ts>GulpR^RNAi1
50 μM
pros^ts> DAPI/Lipid
pros^ts>Tk^RNAi
pros^ts>GulpR^RNAi1
Fluor 488 (AMG)
Tk+ cell (AMG)
pros^ts>
pros^ts>Tk^RNAi
pros^ts>GulpR^RNAi
PMG
pros^ts> DAPI/Tk
pros^ts>Tk^RNAi
pros^ts>GulpR^RNAi1
50 μM
pros^ts> DAPI/Lipid
pros^ts>Tk^RNAi
pros^ts>GulpR^RNAi1
Fluor 488 (PMG)
Tk+ cell (PMG)
ns

S3 Fig: Knockdown of *GulpR* after eclosion decreases Tk+ EECs in the AMG and PMG and increases lipid accumulation in the AMG. Representative micrographs and quantification of Tk+ cell number and lipid accumulation (Fluor 488) in the AMG and PMG of flies with a temperature sensitive prospero driver ($pros^{ts}$>) driving either Tk^{RNAi}or $GulpR^{RNAi}$. Flies were maintained at 21 ^{o}C until eclosion. Expression was activated after eclosion by transferring flies to 29 degrees for 5-8 days. The mean of a minimum of 9 intestines is shown. Scale bar 50 µM. For lipid accumulation, significance was calculated using a one-way ordinary ANOVA with a Dunnett's multiple comparisons test. For Tk+ cells, significance was calculated using a Brown-Forsythe ANOVA with a Dunnett's T3 multiple comparisons test. **** p<0.0001, * p<0.05, ns not significant.
