# Supplementary material for: The Drosophila G protein-coupled receptor, GulpR, is essential for lipid mobilization in response to nutrient-limitation
Source: PLoS Genet. 2025 Dec 12;21(12):e1011982. doi: 10.1371/journal.pgen.1011982 (PMC12711087; doi:10.1371/journal.pgen.1011982)
Supplement: S4 Fig — (PDF) [file pgen.1011982.s004.pdf]

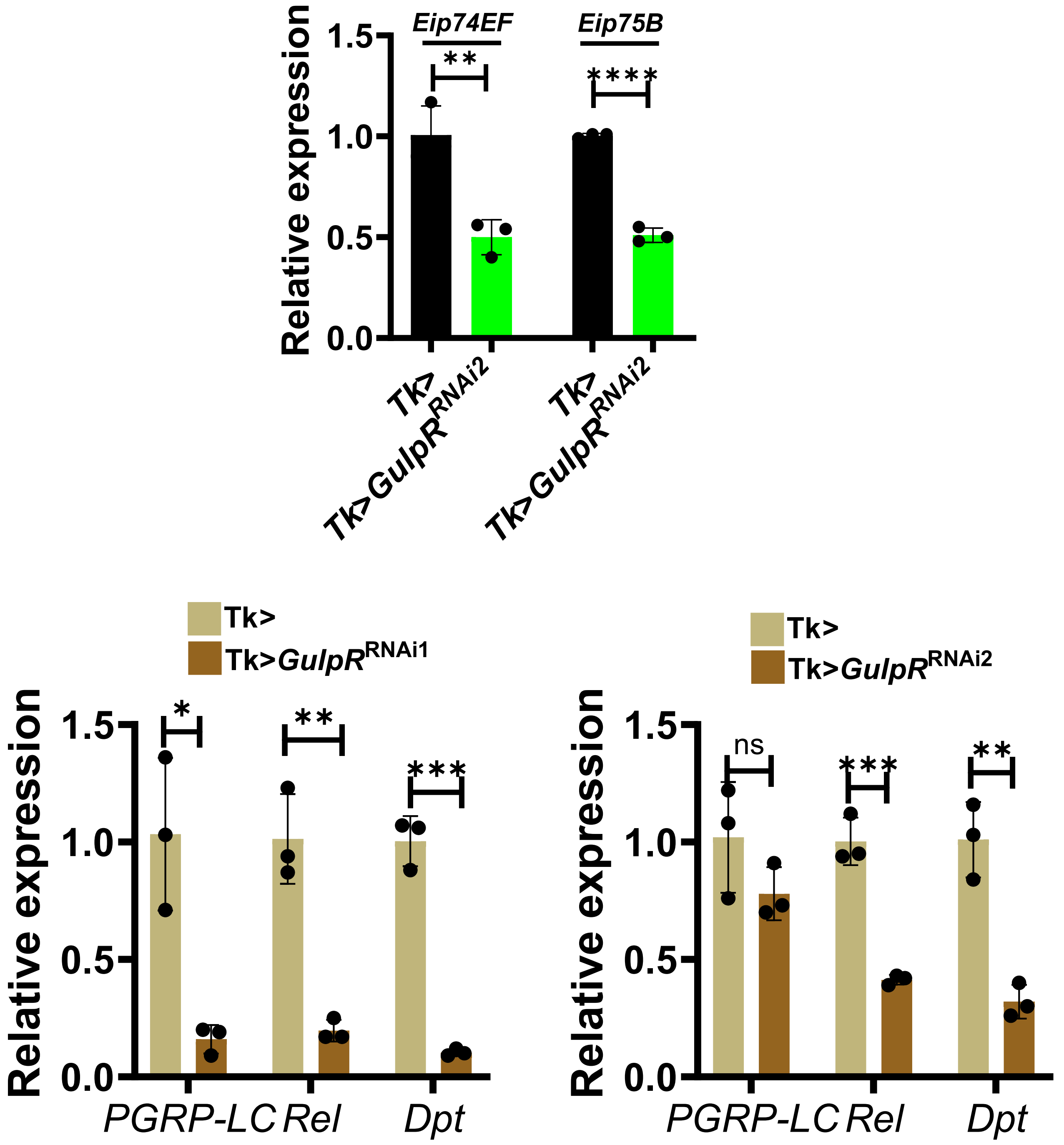

S4 Fig: Knockdown of *GulpR* in Tk+ EECs decreases intestinal ecdysone and IMD signaling. qRT-PCR analysis of the ecdysone-regulated genes *Eip74EF* and *Eip75B* and the IMD-regulated genes *PGRP-LC*, *Rel*, and *Dpt* expression in the intestines of Tk> and Tk>*GulpR*^{RNAi1} and Tk>*Gulp*R^{RNAi2} flies. **** $p<0.0001$, *** $p<0.001$, ** $p<0.01$, * $p<0.05$.
