# Supplementary material for: The Drosophila G protein-coupled receptor, GulpR, is essential for lipid mobilization in response to nutrient-limitation
Source: PLoS Genet. 2025 Dec 12;21(12):e1011982. doi: 10.1371/journal.pgen.1011982 (PMC12711087; doi:10.1371/journal.pgen.1011982)
Supplement: S5 Fig — (PDF) [file pgen.1011982.s005.pdf]

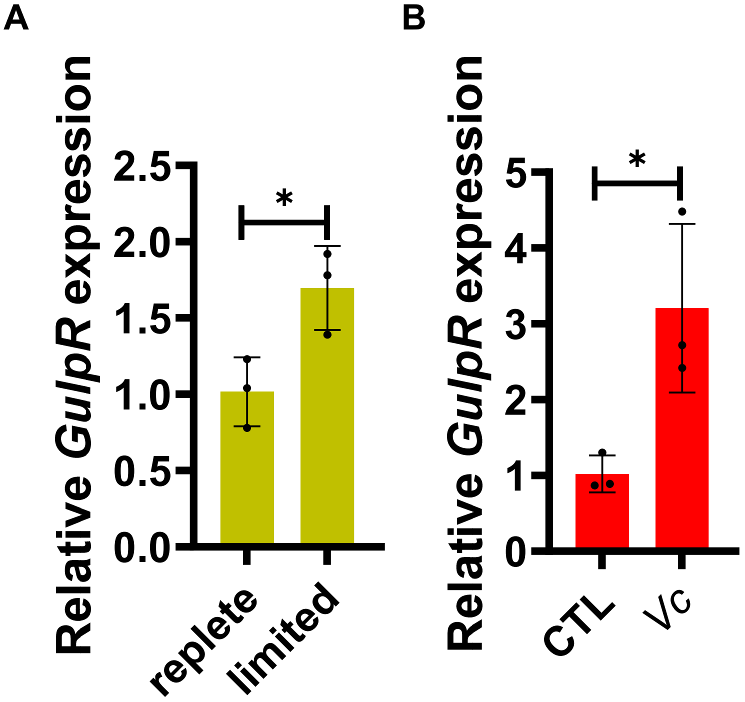

S5 Fig: Both infection and starvation increase expression of GulpR. qRT-PCR of GulpR expression in the intestines of control flies (A) under nutrient-replete or limited conditions or (B) infected with *V. cholerae* (*Vc*) . The mean of biological triplicates is shown. Error bars represent the standard deviation. Significance was calculated using a student's t test* $p<0.05$.
