# Supplementary material for: The Drosophila G protein-coupled receptor, GulpR, is essential for lipid mobilization in response to nutrient-limitation
Source: PLoS Genet. 2025 Dec 12;21(12):e1011982. doi: 10.1371/journal.pgen.1011982 (PMC12711087; doi:10.1371/journal.pgen.1011982)
Supplement: S6 Fig — (PDF) [file pgen.1011982.s006.pdf]

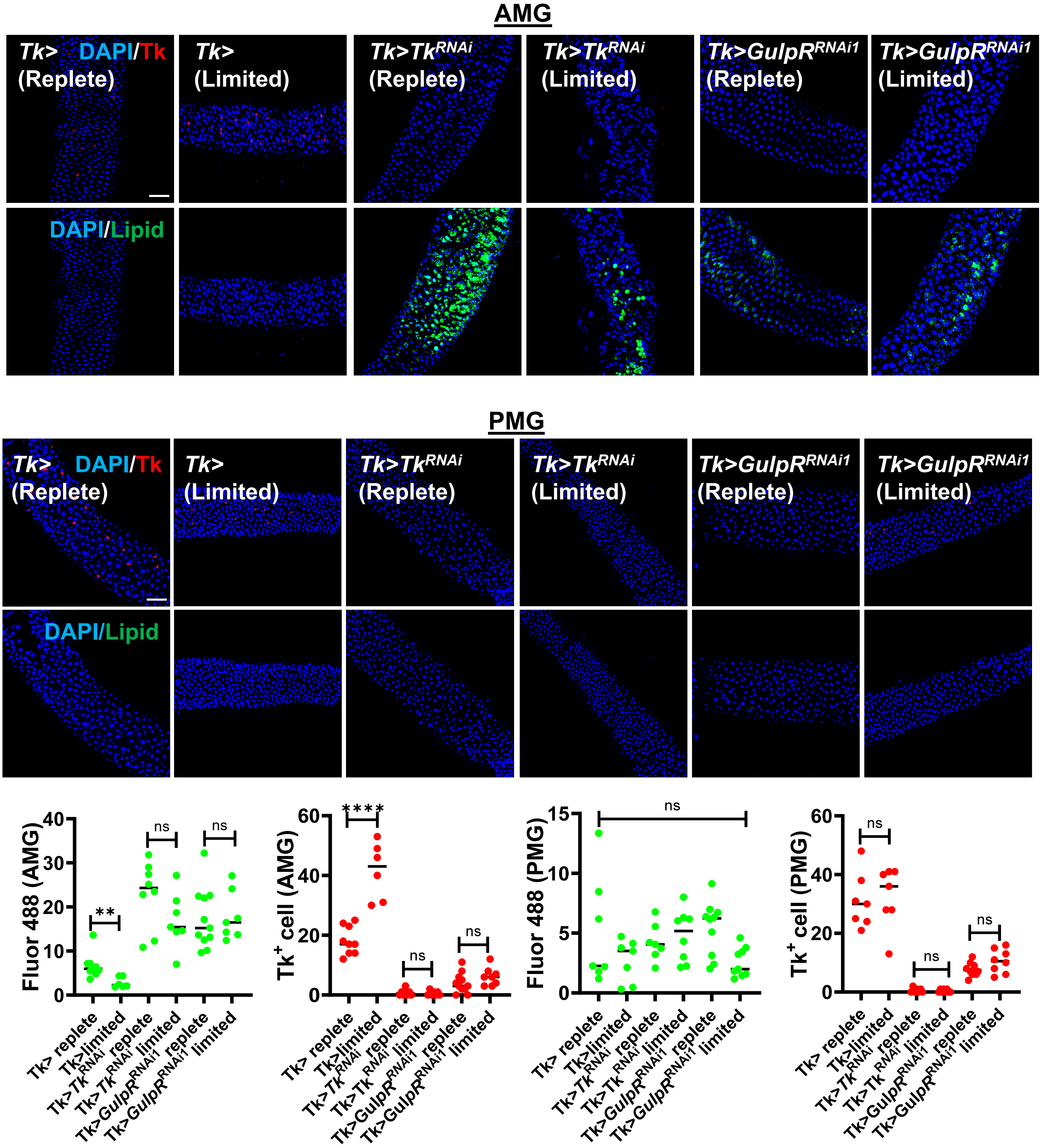

S6 Fig: Knockdown of *GulpR* and *Tk* in Tk+ EECs blocks an increase in Tk+ EECs and utilization of lipids in the AMG during starvation. Representative micrographs and quantification of lipids and Tk+ EECs in the AMG and PMG of flies of the indicated genotype maintained in nutrient-replete or nutrient-limited conditions. Scale bar 50 μM. The mean of a minimum of 6 intestines is shown. Significance was calculated using a student's t test. **** p<0.0001, ns not significant.
