# Supplementary material for: The Drosophila G protein-coupled receptor, GulpR, is essential for lipid mobilization in response to nutrient-limitation
Source: PLoS Genet. 2025 Dec 12;21(12):e1011982. doi: 10.1371/journal.pgen.1011982 (PMC12711087; doi:10.1371/journal.pgen.1011982)
Supplement: S7 Fig — (PDF) [file pgen.1011982.s007.pdf]

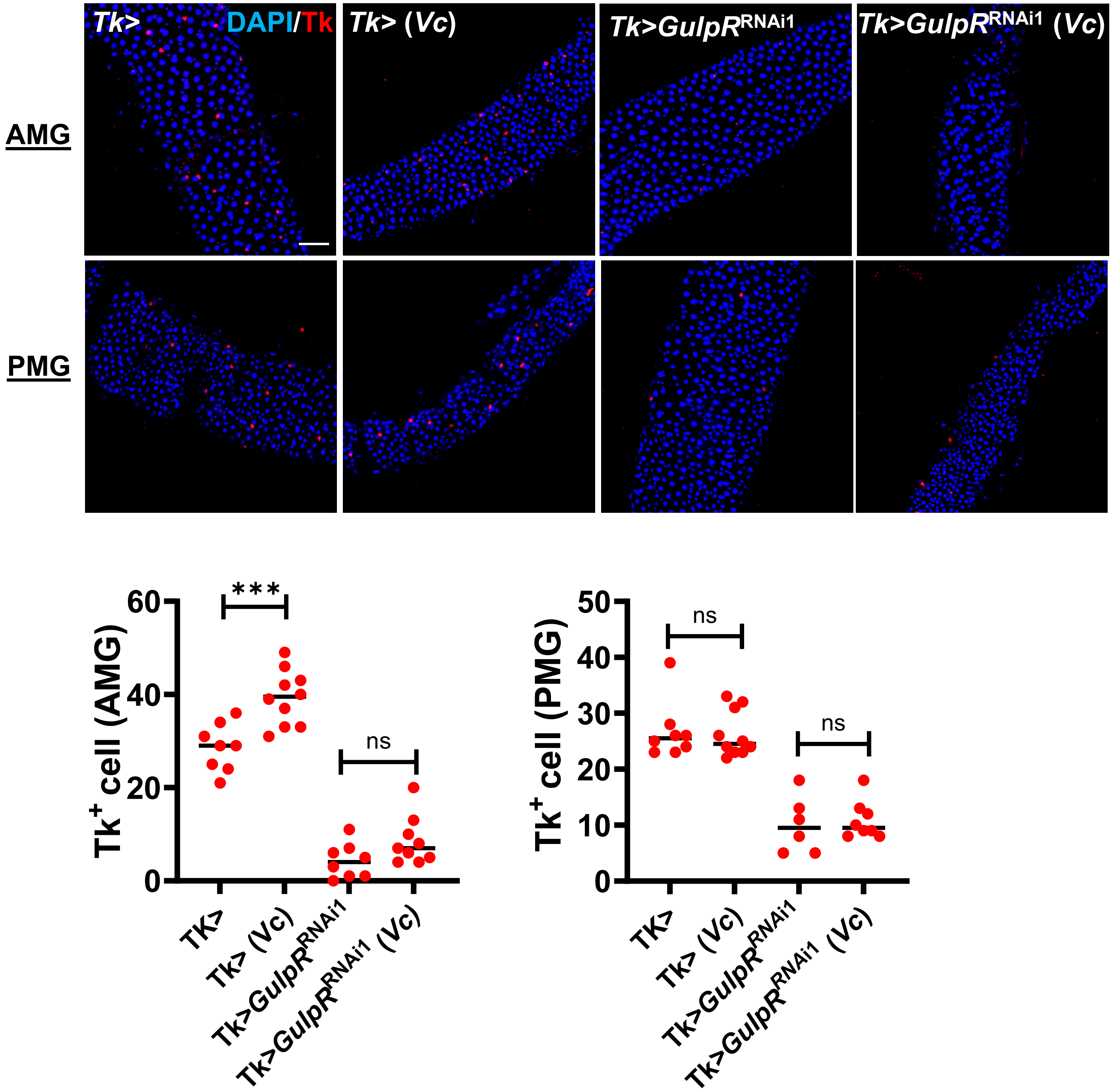

S7 Fig: Knockdown of *GulpR* in Tk+ EECs blocks an increase in Tk+ EECs during *V. cholerae* infection. Representative micrographs and quantification of lipids and Tk+ EECs in the AMG and PMG of flies of the indicated genotype either fed LB alone or LB inoculated with *V. cholerae* (*Vc*). The mean of a minimum of 8 intestines is shown. Scale bar 50 μM. Significance was calculated using a student's t test. *** p<0.001, ns not significant.
